# Nonzero-Sum Time Perception Is Associated with Greater Willingness to Help

**DOI:** 10.3390/ejihpe15050090

**Published:** 2025-05-21

**Authors:** Yu Niiya, Syamil Yakin, Lora E. Park, Ya-Hui Chang

**Affiliations:** 1 Department and Faculty of Global and Interdisciplinary Studies, Hosei University, Ichigaya Campus, Tokyo 102-8160, Japan; 2 Department of Psychology, The Ohio State University, Columbus, OH 43210, USA; 3 Department of Psychology, University at Buffalo, The State University of New York, Buffalo, NY 14260, USA; lorapark@buffalo.edu (L.E.P.);

**Keywords:** nonzero-sum, zero-sum, time perception, helping, prosocial

## Abstract

People are less likely to help others when they view time as a scarce resource. Does changing people’s *perception* of time influence their willingness to help? We hypothesized that people would be more willing to help and would allocate more time to helping others when they view time as a *nonzero-sum* resource (i.e., as a resource that merely exists or that can be created moment-by-moment with their interactions with others) versus a *zero-sum* resource (i.e., a commodity that can be lost, taken, or given away). Study 1 measured people’s perception of time and their willingness to help in hypothetical vignettes. Studies 2 and 3 manipulated the perception of time to examine its effect on people’s willingness to help others and the amount of time they wanted to spend helping. Study 3 further examined prosocial motivation as a potential mediator. Across the three studies, we demonstrated that when people perceive time as a nonzero-sum resource versus a zero-sum resource, people are more willing to help others. People’s prosocial motivation to reduce others’ distress mediated this relationship. We speculate that when people perceive time to be nonzero-sum, time spent helping others is not viewed as costly, but as a resource to invest in to benefit both themselves and others.

## 1. Introduction

Time is a limited, precious resource that people allocate to important activities. Books and workshops on time management offer advice on how to maximize one’s time effectively to make progress on goals in everyday life. Although viewing time as scarce and limited can spur people to take action (Karau & Kelly, 1992), this perception can also reduce the likelihood of helping others (Connelly et al., 2014; Jiang et al., 2024; Škerlavaj et al., 2018). Indeed, one of the strongest predictors of people’s decision to help is the amount of time available and time required for help (Darley & Batson, 1973; Fritzsche et al., 2000). When considering whether to help or not, people may be dissuaded from helping because they perceive that time is limited and that helping would detract from their time. In the present research, we propose that changing people’s *perception* of time—as a nonzero-sum resource—may be one of the key factors to increasing people’s willingness to help and to allocating more time to help others.

### 1.1. Time as Zero-Sum Versus Nonzero-Sum

Time, like money, is a valuable resource that people frequently use in transactions with others; time can be spent, saved, or borrowed (Lakoff & Johnson, 1980). Similar to money, time is often perceived as a zero-sum resource: people tend to believe that spending time on others reduces the time they can spend on themselves and vice versa (Niiya, 2019). For example, when a talkative colleague comes by the office to engage in a seemingly endless chat, one might think that they are taking away one’s time and feel frustrated as a result. In this example, people perceive time as a limited resource that is spent on themselves *or* on others, but not on both. This view reflects a zero-sum perception of time because spending time on others implies less time for oneself and thus incurs a cost to the self.

In contrast, time can be perceived as a nonzero-sum resource that merely exists and cannot be taken away; people can conceive of time as a resource that is created moment-by-moment in their interactions with others, rather than a commodity that can be lost, taken, or given away (Niiya, 2019). For example, when a parent takes a day off from work to accompany their child to a soccer tournament, they may not perceive that their child is taking away their time. Instead, time spent with their child may feel like time spent on themselves, enjoying each other’s company. When time is perceived in this nonzero-sum way, spending time on others also implies that one is spending time on oneself, so spending time on others is not perceived to be costly to the self (Niiya, 2019).^1^

Past research has shown that perceiving time as nonzero-sum is related to increased psychological well-being. For example, nonzero-sum perception of time was associated with greater autonomy, competence, relatedness, satisfaction with life, and lower perceived stress among a sample of Japanese participants (Niiya, 2019). Nonzero-sum time perception was associated with greater relatedness and life satisfaction among U.S. participants as well (Yakin & Niiya, 2023). In an experience-sampling study, participants reported their time perception and psychological well-being at random, five times a day, for seven days. Participants with higher (vs. lower) nonzero-sum time perception reported greater life satisfaction, fulfillment of psychological needs, and lower time pressure (Niiya & Suyama, 2023). Moreover, when participants experienced higher nonzero-sum time perceptions than usual, they reported greater life satisfaction, fulfillment of psychological needs, and lower time pressure relative to their own average.

In contrast, having a zero-sum perception of time showed mixed findings. For example, a zero-sum perception that other people are taking away one’s time and that one is taking away others’ time was associated with decreased autonomy, competence, relatedness, and reduced feelings of time affluence among Japanese (Niiya, 2019; Niiya & Suyama, 2023) and American (Yakin & Niiya, 2023) participants. Another form of zero-sum perception of time—the perception that one is offering time or sacrificing time for others—was either unassociated (Yakin & Niiya, 2023) or negatively associated (Niiya & Suyama, 2023) with psychological well-being and feelings of time affluence. The perception that one is offering time to others may have resulted in mixed findings given that this perception involves both other-focused aspects while also being zero-sum.

### 1.2. Time Perception and Willingness to Help

To date, no studies have examined whether having a zero-sum or nonzero-sum perception of time has consequences beyond the self, such as willingness to help others. In the present research, we investigated whether people’s perception of time as zero-sum versus non-zero sum would differentially predict prosocial motives and in turn, their willingness to help others.

When deciding whether to help—and how much to help—others, people often weigh the costs of helping (Dovidio et al., 1991). We hypothesized that in helping situations, people who perceive time as zero-sum would be more hesitant to provide help, because they view time spent helping others as costly to themselves. Consistent with this idea, previous research found that activating a zero-sum mindset decreased helping and cooperation (Andrews Fearon & Götz, 2024); for example, the zero-sum belief that one person’s success comes at the expense of another person’s failure reduced willingness to help at work (Kakkar & Sivanathan, 2022; Sirola & Pitesa, 2017) and decreased willingness to help a colleague learn how to succeed on their own (Chernyak-Hai & Davidai, 2022). Thus, people were unwilling to help others succeed when this undermined their own chance of success.

If zero-sum perceptions are related to less prosocial tendencies, might having a *nonzero-sum* perception of time increase people’s willingness to help others? When people perceive time as nonzero-sum, they may be more willing to help because spending time on others does not reduce the time they could otherwise spend on themselves. That is, for those with a nonzero-sum view of time, spending time on others may feel like they are simultaneously spending time on themselves. Because helping does not incur any time costs to the self, people with a nonzero-sum view of time may not need to weigh the cost against the benefit: they may simply choose to help based on how beneficial helping is to others rather than on how costly helping is to themselves or whether they expect compensation for their time cost (Clark et al., 1986; Li & Hui, 2019).

Moreover, seeing time as a nonzero-sum resource may be associated with a feeling of interdependence between the recipient and provider of help. Whereas a zero-sum mindset was found to be associated with seeing the world as less interconnected (Andrews Fearon & Götz, 2024), a nonzero-sum perception of time may be related to the belief that what is good for oneself and what is good for another person are interrelated. Along these lines, research has shown that interdependence fosters prosocial and cooperative behavior (e.g., Columbus & Molho, 2022; Columbus et al., 2021). For example, an experience sampling study found that when people perceive mutual dependence with a partner in a given situation, they report behaving more cooperatively and their partners also perceive them to behave cooperatively (Columbus et al., 2021). Furthermore, research has shown that people with compassionate goals to support others’ well-being are more likely to have a nonzero-sum belief about relationships (i.e., a belief that relationship problems can be solved in mutually beneficial ways), which predicts greater responsiveness toward others (Crocker et al., 2017) and greater willingness to help a stranger or colleague at work (Niiya & Yakin, 2024). Based on such findings, we propose that prosocial motives would mediate the relationship between nonzero-sum perception of time and willingness to help.

### 1.3. Overview of Present Research

The current studies expand upon previous research in three key ways. First, we examined whether having a nonzero-sum (vs. zero-sum) perception of time would differentially predict willingness to spend time helping others. Specifically, we sought to distinguish between perceiving time as nonzero-sum from the perception that one is offering time to others. Offering time to others presents an interesting case, because while it reflects a mindset of zero-sum perception of time, it is also likely to be related to prosocial motives. For example, when one spends time listening to their friend complain about their boss, they may perceive that they are offering or sacrificing time for their friend. The motivation for spending time on their friend may be prosocial, but the perception that one is offering or sacrificing time implies that they see time as zero-sum. In the present research, we compared the effect of nonzero-sum time perception versus the perception that one is offering or sacrificing time for others to show that it is the nonzero-sum vs. zero-sum distinction that produces different helping outcomes rather than prosocial orientation. We predicted that offering time to others may reveal a weaker association with willingness to help than nonzero-sum time perceptions because offering time reflects a zero-sum time perception, despite being prosocial. Helping is not purely altruistic and people are likely to consider the costs of helping in their decision to help (Dovidio et al., 1991). People may think that by helping others they are reducing their own time resources and may therefore be stingier in allocating their time to help others, compared to when they perceive time to be nonzero-sum.

Second, we examined potential mediators of the proposed relationships; specifically, we tested whether prosocial motives accounted for the relationship between nonzero-sum time perceptions and willingness to help. We theorized that people who perceive time as nonzero-sum do not need to worry about depleting their time resources in helping others. If they are not concerned about costs to the self, they might be more willing to help others via increased motivation to benefit others, rather than a motivation to benefit themselves (Clark et al., 1986). We focused on two types of prosocial motivations that were examined in past research on prosocial spending: recipient-support motives (i.e., to reduce others’ distress and help solve their problems) and recipient-enhancement motives (i.e., to increase others’ well-being; Caprariello & Reis, 2021). Past research found that when people were motivated by others’ benefits versus self-benefits, prosocial spending was associated with greater recipient-support and recipient-enhancement motives, which resulted in greater hedonic and eudaemonic well-being (Caprariello & Reis, 2021). Although nonzero-sum time perception is likely to predict both prosocial motives, we expected recipient-support motives to play a stronger mediating role given that helping in the current study consisted of lending a hand to a colleague who was experiencing trouble with their assigned work. Because the colleague was in distress, we expected that in such contexts, people would experience empathic concerns and recipient-support motives that would be related to a desire to reduce the colleague’s distress, more so than seeking to enhance their happiness.

Third, in contrast to previous studies that focused on cross-sectional associations between time perceptions and well-being, we experimentally manipulated time perceptions to investigate their causal effects on willingness to help. Although Niiya and Suyama (2023) found that time perception varied within individuals, the current research is the first to test whether *changing* people’s perceptions of time directly influences their willingness to help others.

We conducted three studies: Study 1 examined whether perceptions of time as a nonzero-sum resource would be associated with greater willingness to help a colleague or friend across various situations. We predicted that having a nonzero-sum perception of time would be related to greater willingness to spend time helping others, whereas zero-sum time perceptions would be related to less willingness to help. Study 2 sought to establish causality by manipulating time perceptions and seeing whether activating a nonzero-sum perception of time would lead to greater helping toward a colleague at work than zero-sum time perceptions (i.e., the perception of time being taken away or offering time to others). Finally, Study 3 added a control condition to test whether the manipulation of nonzero-sum time increased perceptions of time as nonzero-sum, whether the time-taken-away and offering time manipulations reduced perceptions of nonzero-sum time, or both. Study 3 also considered prosocial motives as potential mediators of the relationship between nonzero-sum time perceptions and helping.

## 2. Method

All datasets, codes, and study materials in the current studies are available at the Open Science Framework (OSF) at https://osf.io/qsa3g/?view_only=fd718de513d744c28a40bec9dda7d073 (accessed on 1 May 2025). Study 1 was approved by the Ohio State University Institutional Review Board (2021E0746). Studies 2 and 3 were reviewed and approved by the University at Buffalo Institutional Review Board (FWA00008824). These studies were not pre-registered. In all studies, participants saw the consent form on the first page of the survey and indicated whether they agreed to proceed with the study.

### 2.1. Participants

#### 2.1.1. Study 1

We recruited 325 participants in the United States (U.S.) through Amazon’s Mechanical Turk as part of a larger study on time perceptions.^2^ Given the importance of the vignettes to the measured variables, 136 participants were excluded for failing one or more attention checks.^3^ The final sample included 189 participants (47% women) who were 82% White, 8.5% Black, 4.2% Asian, 1.1% American Indian/Native, and 4.2% other ethnicities ranging from 22 to 72 years of age (*M* = 39.63, *SD* = 11.14). A post hoc power analysis using G*Power 3.1.9.7 showed that with *n* = 189 and α = 0.05, we had a power of 0.86 to detect an effect of f^2^ = 0.04 in a regression with three predictors. Data collection was conducted in 2019 before the COVID-19 pandemic.

#### 2.1.2. Study 2

We recruited undergraduate participants from the introductory psychology subject pool at a large university in the midwestern U.S. in exchange for course credit. G*Power 3.1.9.7 indicated that at least 390 participants were needed to ensure 95% statistical power to detect an effect size of 0.20 when comparing three groups in ANOVA. A total of 480 participants started the online study, but two failed to complete the manipulation and seven did not complete the main dependent variables, leaving a final sample of 471 participants (45.6% women), including 59.0% White, 10.8% Black, 21.7% Asian, 1.3% American Indian/Native, and 6.8% other ethnicity participants ranging from 18 to 59 years of age (*M* = 19.19, *SD* = 2.33). Data collection was conducted in 2021 during a period when the U.S. was seeing a rapid spread of COVID-19.^4^

#### 2.1.3. Study 3

We recruited participants from a wider age range instead of undergraduates. Using G*Power 3.1.9.7, we determined that at least 436 participants needed to have 95% power to detect an effect size of 0.20 when comparing four groups in ANOVA. We recruited 535 participants from Research Match, but one person dropped out before completing the dependent variables, leaving a final sample of 534 (76.2% women), including 91.6% White, 4.1% Black, 3.9% Asian, 1.1% American Indian/Native, and 2.2% other ethnicity participants ranging from 19 to 91 years of age (*M* = 53.40, *SD* = 16.53). Data collection was conducted in 2022 when the spread of COVID-19 was relatively low.

### 2.2. Procedure

#### 2.2.1. Study 1

Participants completed the time perception scale and then read three vignettes and indicated how much of their free time they were willing to spend helping a friend or colleague at work, followed by attention check items.

**Time perception.** Participants responded to the Time Perception Scale (Niiya, 2019; see Yakin & Niiya, 2023 for scale validation in the U.S.) by rating how often they felt various ways in their daily life on a scale from 1 = *never* to 6 = *always*. The items consisted of nonzero-sum time perceptions (e.g., “I feel that the time I spend on others is time spent on myself;” 3 items, α = 0.78), and zero-sum time perceptions of offering time (e.g., “I feel that I am giving away my time to others;” 3 items, α = 0.68), and time being taken away (e.g., “I feel that others are stealing my time;” 2 items, Spearman–Brown’s ρ = 0.70, *p* < 0.001; Eisinga et al., 2013).^5^

**Helping time.** Participants were presented with three vignettes (in randomized order) in which they had a limited amount of free time to use for themselves and/or for helping a friend or colleague. The scenarios involved the following: (a) helping cover for a colleague at work who was late for their shift; (b) running errands for a sick friend; and (c) helping a colleague who was behind in their assigned work (see Appendix A). Participants used a slider scale from 0 to the maximum amount of available free time in each vignette (e.g., 240 min if the vignette said participants had 4 h of free time) to indicate how much of their free time they intended to allocate to help their friend or colleague. Because each vignette had a different amount of available free time (i.e., 2 to 4 h), we converted the helping time measure to percentages and then computed their means (α = 0.76).

#### 2.2.2. Study 2

Participants were randomly assigned to write about an experience in which they felt that (a) others (e.g., family member, friend, partner, acquaintance, someone else) were taking away their time (*time-taken-away*); (b) they were offering time to others (*offering time*); or (c) time spent on others was time spent on themselves (*nonzero-sum time*). In all conditions, participants were asked to spend at least 2 min writing a detailed memory of their experience in a textbox before they could continue with the survey. Participants then responded to the Time Perception Scale (Niiya, 2019; Yakin & Niiya, 2023) which served as a manipulation check. Specifically, they rated their perception of time-taken-away (ρ = 0.70, *p* < 0.001), offering time (α = 0.81), and nonzero-sum time (α = 0.67) from 1 = *never* to 6 = *always*.

Next, all participants read one vignette from Study 1 in which they imagined having 4 h of free time to use for themselves and/or to help their friend at work. Participants reported how many minutes they would allocate to helping their friend using a sliding scale from 0 to 240 min. Finally, participants answered two questions indicating how willing they would be to offer help (“How much would you want to help your friend?” and “How much would you hesitate to help your friend?” *reversed*) from 1 = *not at all* to 7 = *very much*. We computed the average of the two items as an index of willingness to help (ρ = 0.36, *p* < 0.001).^6^

#### 2.2.3. Study 3

Participants were randomly assigned to one of four conditions: perception of nonzero-sum time, time-taken-away, offering time, or a control condition. For each condition, participants were asked to recall an experience in which they felt that time spent on their friend was time spent on themselves (nonzero-sum time condition), their friend was taking away their time (time-taken-away condition), they were offering time to their friend (offering time condition), or about their experience of waking up the previous morning (control). We sought to strengthen the manipulation by instructing all participants to write about an instance involving a friend to reduce variability in the perceived closeness of the relationship that was recalled in the nonzero-sum and zero-sum time conditions. For all conditions, participants were asked to spend at least 2 min writing a detailed memory of their experience before continuing. Following the writing task, participants completed the same Time Perception Scale (Niiya, 2019; Yakin & Niiya, 2023) as before as a manipulation check (time-taken-away: ρ = 0.87, *p* < 0.001, offering time: α = 0.78, nonzero-sum time: α = 0.81).

Next, participants read the same vignette as in Study 2 and, using the same response scales as before, rated their willingness to help (ρ = 0.52, *p* < 0.001) and the amount of time they would allocate to helping their colleague (“Jeff”). In addition, they completed the following measures.

**Prosocial motives.** Participants responded to 11 items—adapted from Caprariello and Reis’ (2021) measure of prosocial motives for spending money—to assess motives for helping the friend in the vignette (e.g., “I wanted to buy something that __ wanted” was changed to “I wanted to give the help that Jeff wanted”). Five items described reasons for helping that focused on *recipient support* (e.g., “Jeff’s concern felt like part of my own, so I am helping out” and “I did not want Jeff to worry” α = 0.86). Six items described reasons for helping that focused on *recipient enhancement* (e.g., “I wanted to give the help that Jeff wanted” and “I valued Jeff’s happiness greatly at the time” α = 0.78). Participants rated how much each item described their reason for helping Jeff on a scale from 1 = *not at all* to 6 = *extremely*.

**Relationship closeness.** We also measured relationship closeness to test the alternative possibility that the nonzero-sum manipulation might be associated with greater helping merely because people felt closer to the target. Participants were shown seven images of two circles (adapted from Aron et al., 1992) with one circle labeled “Self” and the other circle labeled “Other” that varied in the degree of overlap. Participants chose the image that best described their relationship with the person in the vignette, which was coded from 1 (*two circles were completely separate from each other*) to 7 (*two circles completely overlapped with each other*) as a measure of relationship closeness.

## 3. Results

### 3.1. Study 1

First, we examined the correlations among variables. Similar to Niiya’s (2019) study, perceptions of time as nonzero-sum were strongly related to perceptions of offering time (*r* = 0.60, *p* < 0.001). However, the perception that time was being taken away was more strongly related to offering time (*r* = 0.49, *p* < 0.001) than a nonzero-sum time perception (*r* = 0.29, *p* < 0.001; *z* = 2.99, *p* = 0.003), suggesting that offering time reflects a zero-sum perception of time (see Supplemental Table S1 for descriptive statistics and correlations among the variables).

Next, we simultaneously regressed helping time on perceptions of time being taken away, offering time, and nonzero-sum time. The regression model was significant, *F*(3, 185) = 3.25, *p* = 0.023; adjusted R^2^ = 0.035. Nonzero-sum time perception significantly predicted greater helping, b = 5.25, SE = 1.93, *p* = 0.01; 95% CI [1.44, 9.01], whereas perceptions of time being taken away, b = −1.44, SE = 1.46, *p* = 0.32; 95% CI [−4.09, 1.55], and offering time did not, b = −0.57, SE = 2.26, *p* = 0.80; 95% CI [−5.16, 3.98]. Including age and gender as covariates did not change the results. Nonzero-sum time perception continued to be significantly associated with greater helping, b = 5.46, SE = 1.93, *p* = 0.005; 95% CI [1.65, 9.27], while time being taken away, b = −1.42, SE = 1.45, *p* = 0.33; 95% CI [−4.27, 1.44], and offering time remained not significant, b = −0.47, SE = 2.27, *p* = 0.84; 95% CI [−4.95, 4.01]. We also conducted separate analyses by vignettes. Despite some slight variations in findings across vignettes, nonzero-sum time perception was consistently, positively associated with helping time in the first (b = 6.95, SE = 3.07, *p* = 0.025, 95% CI [0.89, 13.01]), second (b = 5.20, SE = 2.67, *p* = 0.053, 95% CI [−0.08, 10.47]) and third vignettes, b = 13.50, SE = 5.44, *p* = 0.014, 95% CI [2.77, 24.23].

Overall, consistent with the hypotheses, Study 1 showed that having a nonzero-sum perception of time was positively related to intentions to spend time helping others. Unexpectedly, perceiving time as zero-sum (i.e., perceptions of time being taken away or offering time) was unrelated to willingness to help. Perhaps people who perceive the time costs of helping are reluctant to help, but may choose to help when they expect that the benefits of helping (e.g., appreciation, reciprocity, self-esteem) can compensate for the time costs.

### 3.2. Study 2

Preliminary analyses showed that the experimental manipulation successfully activated participants’ nonzero-sum time perception (see Supplemental Figure S1 and Table S2 for descriptive statistics and correlations of the variables). To test the effectiveness of the manipulation, we conducted a MANOVA with condition as the independent variable and nonzero-sum time, offering time, and time-taken-away as the dependent variables. The multivariate test showed a significant main effect of condition, *F*(5, 934) = 5.98, *p* < 0.001; η_p_^2^ = 0.04. The effect of condition was significant for nonzero-sum time, *F*(2, 468) = 6.14, *p* = 0.002; η_p_^2^ = 0.03, and time-taken-away, *F*(2, 468) = 12.02, *p* < 0.001; η_p_^2^ = 0.05, but not for offering time, *F*(2, 468) = 0.32, *p* = 0.73; η_p_^2^ = 0.001. Contrast tests revealed that participants in the nonzero-sum time condition reported significantly higher nonzero-sum time perceptions (*M* = 3.30, *SD* = 0.72) than those in the offering time (*M* = 3.05, *SD* = 0.65, *p* = 0.002) and time-taken-away conditions (*M* = 3.07, *SD* = 0.74, *p* = 0.004). Contrast tests also revealed that participants in the time-taken-away condition reported significantly higher perceptions that time was taken away (*M* = 2.88, *SD* = 0.86) than those in the nonzero-sum (*M* = 2.40, *SD* = 0.89, *p* < 0.001) and offering time conditions (*M* = 2.68, *SD* = 0.81, *p* = 0.043). However, participants did not differ significantly in their perceptions of offering time across conditions (*M*_nonzero-sum_ = 3.52, *SD* = 0.78; *M*_offering time_ = 3.46, *SD* = 0.74; *M*_time-taken-away_ *=* 3.46, *SD* = 0.80, *p*s > 0.48). Overall, the manipulations for nonzero-sum time and time-taken-away conditions were successful, but not the manipulation for offering time.

**Figure 1:**
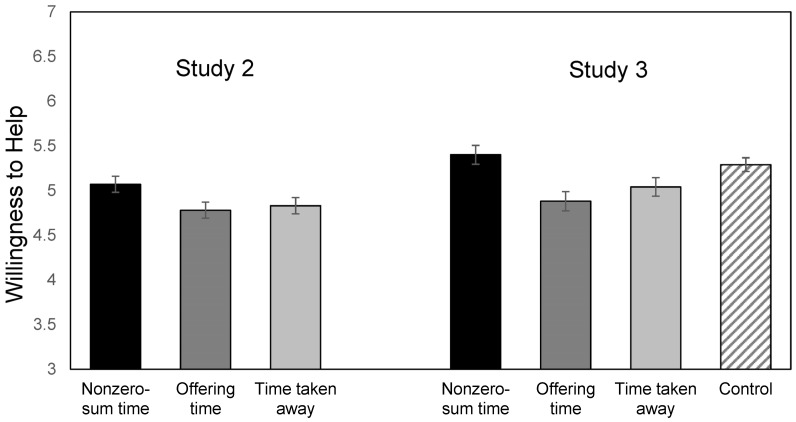
Willingness to help by time perception condition (Studies 2 and 3). Means are reported with +1 and −1 standard error bars.

Next, to test our primary hypotheses, we examined the effect of condition on willingness to help (see Figure 1 left panel). As expected, an ANOVA with condition as the independent variable and willingness to help as the dependent variable showed a significant main effect of condition, *F*(2, 468) = 3.16, *p* = 0.043; η_p_^2^ = 0.01. Contrast tests revealed that participants in the nonzero-sum time condition were more willing to help (*M* = 5.07, *SD* = 1.12) than those in the offering time condition (*M* = 4.78, *SD* = 1.11, *F*(468) = 5.62, *p* = 0.018, η_p_^2^ = 0.012) and slightly more willing to help than those in the time-taken-away condition (*M* = 4.82, *SD* = 1.06, *F*(468) = 3.73, *p* = 0.054, η_p_^2^ = 0.01).

**Figure 2:**
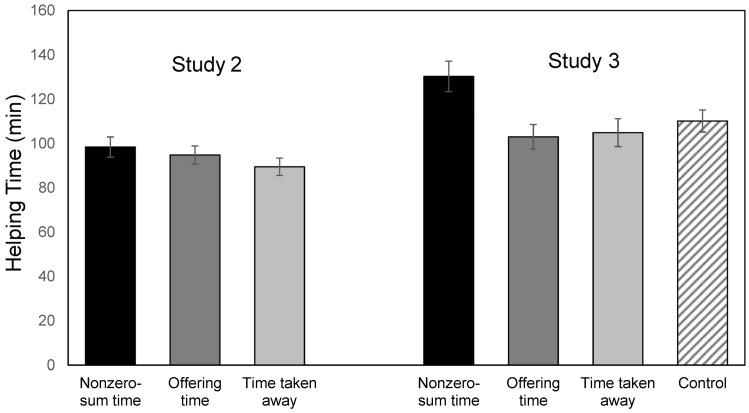
Time allocated to helping by time perception condition (Studies 2 and 3). Means are reported with +1 and −1 standard error bars.

An ANOVA with time allocated to helping as the dependent variable and condition as the independent variable showed that the main effect of condition was not significant, *F*(2, 468) = 1.11; *p* = 0.33. Contrast tests revealed that the amount of time participants in the nonzero-sum condition intended to spend on helping did not differ from those in the offering time and time-taken-away conditions, *Fs* < 2.20. However, the pattern of results (see Figure 2, left panel) showed that participants in the nonzero-sum condition intended to spend longer time helping (*M* = 98.37, *SD* = 56.38) than those in the offering time condition (*M* = 94.78, *SD* = 53.0) and those in the time-taken-away condition (*M =* 89.45, *SD* = 48.39). However, these results should be interpreted with caution, given that the overall ANOVA was not significant.

Finally, we repeated the above analyses while controlling for age and gender. We confirmed that the inclusion of these covariates did not alter the significance of the main findings. Specifically, the main effect of condition on willingness to help, *F*(2, 465) = 3.55, *p* = 0.030 and η_p_^2^ = 0.02, remained significant while the main effect of condition on time allocated to helping remained non-significant, *F*(3, 465) = 1.05 and *p* = 0.35.

In sum, Study 2 found that the writing task was generally successful in shifting perceptions of time and that people who were prompted to think of time as nonzero-sum were more willing to help a colleague than those who were prompted to think of time as a zero-sum. However, the effects of the manipulation were limited to willingness to help and did not extend to the amount of time intended to help. The null results may be due to the weak manipulation of offering time or to the fact that data were collected from undergraduate students during the semester when participants may have felt constrained by the amount of time that was actually available to them.

### 3.3. Study 3

#### 3.3.1. Manipulation Checks

A MANOVA with condition as the independent variable and nonzero-sum time, offering time, and time-taken-away as the dependent variables showed a significant main effect of condition, *F*(9, 1578) = 65.74, *p* < 0.001; η_p_^2^ = 0.27 (see Supplemental Figure S2).^7^ As shown in Table 1, the tests of between-subject effects showed significant effects of condition on perceptions of nonzero-sum time (*F*(3, 526) = 81.29, *p* < 0.001, η_p_^2^ = 0.32), offering time (*F*(3, 526) = 102.29, *p* < 0.001, η_p_^2^ = 0.37), and time-taken-away (*F*(3, 526) = 117.33, *p* < 0.001, η_p_^2^ = 0.40). As expected, contrast tests revealed that nonzero-sum time perceptions were higher in the nonzero-sum condition than in any other conditions (see Supplemental Table S3). Perceptions of time being taken away were higher in the time-taken-away condition versus the other conditions. Offering time was higher in the offering time condition than the nonzero-sum time and control conditions, and marginally higher than the time-taken-away condition (*p* = 0.07). Overall, these findings suggest that the manipulation of time perception in Study 3 was successful and stronger than in Study 2.

#### 3.3.2. Willingness to Help and Time Spent Helping

As expected, there was a significant main effect of condition on willingness to help, *F*(3, 530) = 5.62, *p* < 0.001 and η_p_^2^ = 0.03 (see Figure 1, right panel, Table 2 for statistics). Consistent with Study 2, contrast tests with nonzero-sum time condition as the reference group revealed that participants in the nonzero-sum time condition were more willing to help their colleagues than those in the offering time and time-taken-away conditions (see Supplemental Table S4). Although the pattern of results showed that participants in the nonzero-sum time condition were more willing to help than those in the control condition, this difference was not significant. Contrast tests with the control condition as the reference group revealed that participants in the offering time and time-taken-away conditions were less willing to help than those in the control condition; the latter finding was marginally significant.

Similar results were found with time allocated to helping (see Figure 2, right panel). In contrast to Study 2, which did not find a significant effect of condition, the results of an ANOVA in the present study showed a significant effect of condition on time allocated to help, *F*(3, 530) = 3.91, *p* = 0.009 and η_p_^2^ = 0.02. Contrast tests with nonzero-sum time condition as the reference group revealed that participants in the nonzero-sum time condition were willing to allocate more time to help than those in the offering time, time-taken-away, or control condition. Contrast tests with the control condition as the reference group showed that participants in the offering time and time-taken-away conditions did not differ significantly from those in the control condition, although their means were in the expected directions (i.e., helping time was lower than those in the control condition).

Together, these findings suggest that activating a nonzero-sum perception of time increased the amount of time allocated to helping (but not their willingness to help), whereas activating a zero-sum perception of time decreased willingness to help (but not time allocated to helping) relative to a control condition.

There was also a main effect of condition on relationship closeness, *F*(3, 530) = 2.63, *p* = 0.050 and η_p_^2^ = 0.02, but when we repeated the above analyses while controlling for relationship closeness, age, and gender, the inclusion of these covariates did not alter the significance of the findings. Specifically, the main effect of condition on willingness to help, *F*(3, 526) = 5.23, *p* = 0.001 and η_p_^2^ = 0.03, and on time allocated to helping, *F*(3, 526) = 3.27, *p* = 0.021 and η_p_^2^ = 0.02, remained significant even after including relationship closeness as a covariate.

The main effect of condition was significant for recipient-support motives (*F*(3, 530) = 3.50, *p* = 0.015, η_p_^2^ = 0.02) but not for recipient enhancement motives, *F*(3, 530) = 2.48, *p* = 0.060, η_p_^2^ = 0.01.

#### 3.3.3. Indirect Effects of Nonzero-Sum Time Manipulation

**Figure 3:**
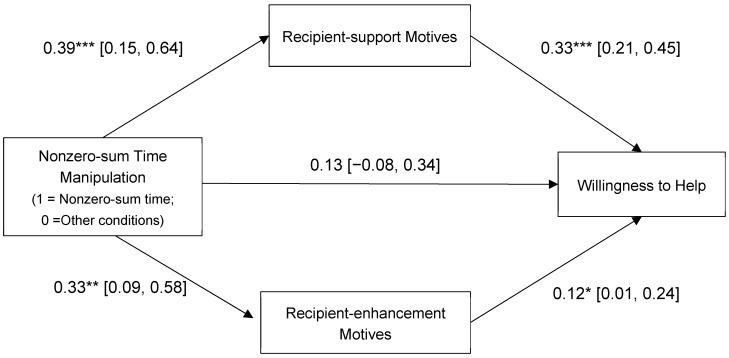
Prosocial motives as mediators of the association between the nonzero-sum time manipulation and willingness to help (Study 3). Indirect effect via recipient-support motives: 0.13, SE = 0.04, 95% CI [0.05, 0.20]; indirect effect via recipient-enhancement motives: 0.04, SE = 0.03, 95% CI [−0.003, 0.11]. Nonzero-sum time manipulation was coded as 1 = nonzero-sum time condition, 0 = offering time condition, 0 = time-taken-away condition; 0 = control. Values reflect unstandardized coefficients. * *p* < 0.05, ** *p* < 0.01, *** *p* < 0.001.

Next, using PROCESS v4.0 model 4 (Hayes, 2022), we conducted a mediation analysis to examine the relationship between the nonzero-sum time manipulation and willingness to help through recipient-support motives and recipient-enhancement motives. The nonzero-sum time manipulation (coded as 1 = nonzero-sum time condition; 0 = offering time condition; 0 = time-taken-away condition; 0 = control) was entered as the independent variable and the two prosocial motives were entered as simultaneous mediators (Figure 3; see Supplemental Table S5 for correlations).

Activating a nonzero-sum perception of time increased recipient-support motives, which were associated with greater willingness to help. Furthermore, the indirect effect through recipient-support motives was significant. Activating a nonzero-sum time perception also increased recipient-enhancement motives, which were associated with greater willingness to help. However, the indirect effect through recipient-enhancement motives was not significant.

**Figure 4:**
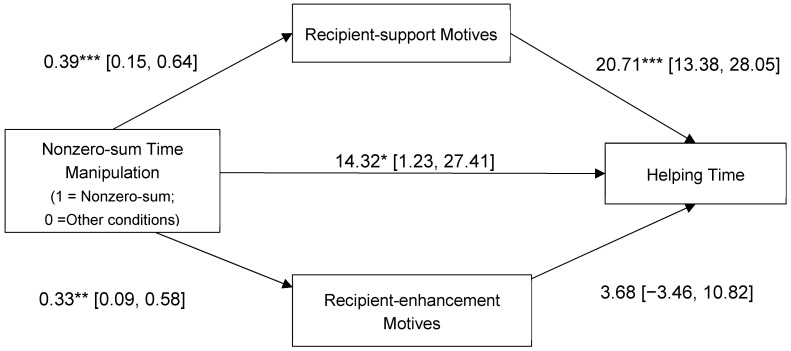
Prosocial motives as mediators of the association between the nonzero-sum time manipulation and time allocated to helping (Study 3). Indirect effect via recipient-support motives: 8.15, SE = 2.88, 95% CI [3.10, 14.19]. Indirect effect via recipient-enhancement motives: 1.23, SE = 1.50, 95% CI [−1.52, 4.52]. Nonzero-sum manipulation was coded as 1 = nonzero-sum time condition, 0 = offering time condition, 0 = time-taken-away condition; 0 = control. Values reflect unstandardized coefficients. * *p* < 0.05, ** *p* < 0.01, *** *p* < 0.001.

Similar results emerged for time allocated to helping (see Figure 4). The nonzero-sum time manipulation had a significant, indirect effect on helping through increased recipient-support motives, but not through recipient-enhancement motives.

Including relationship closeness, age, and gender in the above analyses did not change the results. The indirect effect of nonzero-sum time manipulation through recipient-support motives remained significant for both willingness to help, b = 0.10, SE = 0.04 and 95% CI = [0.02, 0.19], and time allocated to help, b = 5.89, SE = 2.58 and 95% CI = [1.37, 11.38]. In contrast, indirect effects through recipient-enhancement motives remained non-significant for willingness to help, b = 0.02, SE = 0.02 and 95% CI = [−0.01, 0.07], and time allocated to helping, b = 0.11, SE = 0.88 and 95% CI = [−1.80, 2.00].

In sum, when people were led to perceive time as nonzero-sum, they reported greater willingness to help others and wanted to spend more time helping others through increased prosocial motives to reduce the recipient’s distress, but not through the motive to enhance the recipient’s welfare.

#### 3.3.4. Indirect Effects of Offering Time on Helping

To see whether the above effects were unique to nonzero-sum time perceptions, we repeated the mediation analysis with offering time as the independent variable. The results showed no significant indirect effects of the offering time condition on willingness to help through recipient-support motives or recipient-enhancement motives (see Supplemental Figure S3). Similar results emerged when we conducted mediation analysis with time allocated to helping as the outcome variable (see Supplemental Figure S4). There were no significant indirect effects of offering time manipulation on helping time through recipient-support motives or recipient-enhancement motives. Overall, these results suggest that although offering time and nonzero-sum time may both appear to be prosocial, only nonzero-sum time perception was associated with greater helping intentions and allocation of time to help others through the motive to reduce the other person’s distress.

## 4. General Discussion

People’s helping decisions often rely on perceived costs to helping (Dovidio et al., 1991; Piliavin, 1991) and in particular, on the amount of time needed to provide the help (Fritzsche et al., 2000). Although past research suggests that time scarcity reduces helping (Connelly et al., 2014; Darley & Batson, 1973; Jiang et al., 2024; Škerlavaj et al., 2018), we predicted that people would be more likely to help others if they perceived that helping did not detract from their own time. Consistent with this idea, we found that perceptions of time as nonzero-sum were related to greater willingness to help.

Specifically, people who viewed time as nonzero-sum allocated more time to helping a friend or colleague (Study 1), and those who were led to recall a past experience when time was perceived as nonzero-sum (vs. zero-sum) showed greater willingness to help their friend (Studies 2 and 3) and allocated more time to helping them (Study 3). Importantly, participants who were led to perceive time as nonzero-sum allocated more time to helping others than those in a control condition, and this association was due to increased prosocial motives to reduce other’s distress (Study 3).

### 4.1. Nonzero-Sum Time Perception and Helping

Our results suggest that spending time helping others may feel less costly when people perceive time as a nonzero-sum versus zero-sum resource. Such findings align with past research on zero-sum beliefs, which showed that when people believe that one person’s success comes at the expense of another person’s failure, they are less likely to help others (Chernyak-Hai & Davidai, 2022; Kakkar & Sivanathan, 2022; Sirola & Pitesa, 2017). Similarly, when people perceive time or success as zero-sum, they may view others as competitors who seek to take away valuable resources, such as time or opportunities for success. Such perceptions may spur people to become cautious about who to help, how much to help, and under what circumstances to help.

In contrast, when people perceive time as nonzero-sum, they may find themselves to be more interdependent and less concerned about protecting their own resources, which may allow them to pay more attention to others’ needs. Indeed, activating a nonzero-sum perception of time increased people’s willingness to help and time allocated to help through increased prosocial motives to alleviate others’ distress. These findings held even after accounting for relationship closeness, suggesting that the activation of nonzero-sum time perceptions did not increase the willingness to help simply by perceiving others to be closer. When people perceive time as nonzero-sum, they may be better able to focus on others’ needs, rather than on how costly the help is or whether they should be compensated through reciprocity.

### 4.2. Perceptions of Offering Time and Helping

In contrast to nonzero-sum time perceptions, the perception of offering time to others or of time being taken away showed no significant associations with time allocated to help (Studies 1 and 3) and decreased willingness to help compared to a control condition (Study 3). Although perceptions of offering time to others generally reflect prosocial motives, the findings of the current studies were similar to those of time being taken away. That is, when people perceived they were offering or sacrificing time for others, they were still perceiving time as zero-sum—a resource that can be diminished by spending it on others. This zero-sum perception may have led participants to view helping as costly and thereby limited how much time they were willing to spend helping others. Participants may have also been concerned about whether the recipient of help would reciprocate the help, and may therefore not have been motivated by prosocial motives to support others.

The present findings differ from past research that found that offering time to others can increase feelings of time affluence (Mogilner et al., 2012). In previous work, participants who were prompted to spend time helping others felt they had *more* spare time than those who wasted their time on an inconsequential task, or those who spent time on themselves. However, the “offering time” instructions in these prior studies did not necessarily require participants to sacrifice their own time for others. Thus, it seems possible that participants in Mogilner et al.’s (2012) studies experienced time affluence because they perceived time as nonzero-sum while spending time helping others.

In sum, while spending time helping others can be described as prosocial, *how* people perceive the time they spend on others (as nonzero-sum or zero-sum) is likely to shape people’s motives to help others, which then influences how they respond to others’ requests for help.

### 4.3. Implications

Whereas past research focused on the link between nonzero-sum time perceptions and psychological well-being (Niiya, 2019; Niiya & Suyama, 2023; Yakin & Niiya, 2023), the current studies are the first to demonstrate that viewing time as nonzero sum has implications beyond the perceiver’s own well-being to increasing prosocial intentions. Past work suggests that prosocial behaviors promote physical and psychological well-being (e.g., Dunn et al., 2014; Eskreis-Winkler et al., 2018; Williamson et al., 2017). Thus, it seems plausible that adopting a nonzero-sum perception of time may improve well-being by boosting helping behavior toward others.

Whereas past studies relied on correlational methods, the present research manipulated time perceptions and found that nonzero-sum time perceptions increased prosocial motives and intentions relative to a zero-sum time and baseline control conditions. Notably, *changing* people’s perceptions of time increased their willingness to help others. Although we only examined the effect of time perception on helping, time perceptions and prosocial outcomes may mutually reinforce each other. For example, people who view time as nonzero-sum may help others more, and consequently, experience greater connectedness with others. Feeling increased connectedness, in turn, might lead individuals to view relationships as more communal, which may then contribute to more helping (Columbus & Molho, 2022; Columbus et al., 2021; Pavey et al., 2011) and perceptions of time as nonzero-sum. Indeed, shifting from zero-sum to nonzero-sum time perceptions may activate communal norms—characterized by noncontingent responsiveness to others’ needs—which has been shown in past work to predict prosocial behavior (Li & Hui, 2019) and satisfaction in close relationships (Clark et al., 1986).

Past research comparing altruism (i.e., trying to increase others’ welfare) and egoism (i.e., trying to increase one’s own welfare; e.g., Batson, 2011) has examined how self-oriented and other-oriented motives predict helping behavior (e.g., Cardador & Wrzesniewski, 2015; Grant & Mayer, 2009). The current studies add to this literature by suggesting that helping is more likely to occur when people perceive helping to be beneficial both for the self and for others, rather than when helping requires self-sacrifice (i.e., when people are only altruistic and offer others their time). This idea resonates with Crocker and Canevello’s (2015) concept of ecosystem motivation in which people realize that fulfilling others’ needs is as important as fulfilling one’s own needs. Activating a nonzero-sum perception of time may thus remind people that it is possible to pursue others’ welfare as well as their own needs simultaneously.

Practically, the present research suggests that changing people’s perception of time relates to increased prosocial tendencies. Attempts to shift people’s perceptions of time could thus facilitate or complement existing interventions that focus primarily on increasing empathy or compassion (e.g., Luberto et al., 2018). Indeed, the manipulation used in the current studies simply asked participants to recall past social interactions in which they viewed time in different ways, and even these brief recollections increased people’s willingness to help someone different from the person they recalled in the manipulation. Such findings suggest that momentarily shifting perceptions of time as a nonzero-sum resource could be an effective strategy to enhance prosociality even when the target of help is unknown.

### 4.4. Limitations and Future Directions

Some findings did not fully align with the hypotheses. As expected, the pattern of means showed that participants in the nonzero-sum time condition in Study 3 were more willing to help than those in the control condition, but these two groups did not differ statistically. Also, in Study 3, participants in the offering time and time-taken-away conditions allocated less time to helping a friend than those in the control condition, but again, these mean differences did not reach statistical significance. Such findings suggest that simultaneously emphasizing nonzero-sum time (while also de-emphasizing zero-sum perception of time) may prove more fruitful in promoting helping than separately manipulating either zero-sum or nonzero-sum time perception. Future research could examine the effects of such interventions relative to a control condition.

We also found support for recipient-support motives as a mediator in explaining the association between nonzero-sum time perception and willingness to help, but the direct effect of nonzero-sum time and helping time remained significant, suggesting that other variables, such as perceived costs in helping or expectation of reciprocity, might account for this link.

We measured intentions to help, rather than actual helping behavior, because the constraints of the COVID-19 pandemic made it difficult to run in-person experiments. The intention to help does not always translate to actual helping behavior (Piliavin, 1991), although the correlation between intention and behavior is usually large (*r* = 0.53; Sheeran, 2002). In particular, normatively controlled intentions, such as intentions based on social desirability concerns, are less predictive of behavior than intentions derived from personal preferences (Sheeran, 2002). In the current research, self-reported willingness to help and allocating time to helping may have been subject to social desirability biases, but we do not think this bias is likely to have altered the main results. Even if social desirability concerns had affected participants’ reports of their helping intentions, they would not explain why we obtained different effects between nonzero-sum time and zero-sum time perceptions. On the surface, perceiving that one is offering time to others seems equally—if not more—socially desirable than perceiving time as nonzero-sum, so it would be difficult to attribute the differing results obtained between these two time perceptions to social desirability concerns. Future research could seek to replicate the current findings in laboratory settings and in the real world and measure actual helping behavior.

The generalizability of our findings is limited in a few ways. In Studies 2 and 3, we used one vignette to measure people’s willingness to help, but the types of situations, relationships, or help may moderate the effect of time perceptions on helping. For example, people may find it easier to perceive time as nonzero-sum when helping a close other versus a distant other. Our vignette also described a situation where helping involved reducing the target’s distress. In Study 3, recipient-support motives—but not recipient-enhancement motives—mediated the association between nonzero-sum perception of time and willingness to help. Future research could examine if recipient-enhancement motives are a stronger mediator if helping involves increasing another person’s happiness and well-being. The current samples were also limited to U.S. participants. Although past research on time perception was conducted with Japanese participants (e.g., Niiya, 2019), we do not know whether the present findings would apply to cultures that have a slower pace of life, such as Mexico or Indonesia (e.g., Levine & Norenzayan, 1999). For example, activating a nonzero-sum time perception might have stronger effects in cultures where people are pressed for time.

We examined willingness to help immediately after the manipulation, so it remains to be seen how long-lasting the effects are of activating different perceptions of time. In an experience sampling study recording participants’ perception of time five times a day for a week, time perception was found to vary throughout the day (Niiya & Suyama, 2023). Based on such findings, we speculate that the effect of the current manipulations of time perceptions may dissipate when participants engage in another task (e.g., task with a deadline) or interact with others (e.g., with someone who takes away their time). Recently, Andrews Fearon and Götz (2024) proposed the idea that people can have a zero-sum mindset that is stable and consistent across domains (e.g., economics, relationships, politics, etc.) and that changing such mindsets may be difficult. Along these lines, we also think that if individuals have a zero-sum mindset, their perception of time may temporarily shift using an intervention like the one in the current studies, but may not change their general zero-sum mindset, which may be more stable over time.

Finally, the present research focused on how participants viewed the time they spent on others—whether they thought it was time taken away, time that they offered, or time that was nonzero-sum. However, it also seems possible that how much time people spent on helping others influences their time perceptions. For example, if one spends 5 min helping a friend, they may perceive it as nonzero-sum, but as they continue spending more time on helping, they may perceive time as being taken away. Perhaps people differ in how much time they think they can afford to spend on others before they begin to see time as zero-sum. This is an intriguing idea that could be investigated in future research.

## 5. Conclusions

Time is often viewed as a scarce resource, and people may be deterred from helping others because of this perception. The current research shows that by altering perceptions of time, people can be nudged to help others. In particular, when people view time as nonzero-sum, time spent helping others may not seem like a cost they have to pay, but a resource they are investing in for themselves and for others. This nonzero-sum time perception seems to prompt people to base their helping decision on the benefits that helping provides to others in need rather than on how much time cost they would have to pay to provide the help.

## Figures and Tables

**Table 1:** Descriptive statistics and results of ANOVAs of the manipulation checks in Study 3 (*n* = 530). Four participants did not complete the time perception scale. The asterix denotes whether the means were statistically different from those of the reference group (in bold) in a contrast test (*** *p* < 0.001; ** *p* < 0.01). The full results of the contrast tests are in Supplemental Table S3.

	Condition			
	Nonzero-Sum Time (*n* = 109)	Offering Time (*n* = 122)	Time-Taken-Away (*n* = 118)	Control (*n* = 181) ^1^
Nonzero-sum time	**4.17** (0.89)	2.50 *** (1.05)	2.46 *** (1.11)	2.22 *** (1.20)
Offering time	3.10 *** (1.18)	**3.86** (0.89)	3.61 (0.99)	1.95 *** (1.08)
Time-taken-away	1.28 *** (0.69)	3.07 ** (1.43)	**3.47** (1.25)	1.56 *** (1.02)

^1^ We randomly assigned participants to one of the four conditions, but more participants in the control condition completed the study, presumably because the essay task was somewhat easier than those in the other conditions.

**Table 2:** Descriptive statistics and results of ANOVAs of the main variables in Study 3 (*n* = 534). The asterix denotes whether the means were statistically different from those of the reference group (the nonzero-sum time condition) in a contrast test (*** *p* < 0.001; ** *p* < 0.01; * *p* < 0.05). The full results of the contrast tests are in Supplemental Table S4.

	Condition			
	Nonzero-Sum Time (*n* = 110)	Offering Time (*n* = 123)	Time-Taken-Away (*n* = 120)	Control (*n* = 181) ^1^
Willingness to help	5.40 (1.11)	4.88 *** (1.19)	5.05 * (1.14)	5.29 (1.03)
Time allocated to help	130.27 (72.33)	102.97 ** (61.87)	104.89 ** (68.96)	110.14 * (66.98)
Recipient-enhancement motives	4.76 (1.11)	4.39 * (1.12)	4.49 (1.31)	4.40 * (1.18)
Recipient-support motives	5.33 (1.03)	4.93 ** (1.08)	4.99 * (1.20)	4.92 ** (1.23)
Relationship closeness	3.46 (1.41)	3.07 * (1.25)	3.26 (1.31)	3.08 * (1.29)

^1^ We randomly assigned participants to one of the four conditions, but more participants in the control condition completed the study, presumably because the essay task was somewhat easier than those in the other conditions.

## Data Availability

All datasets, codes, and study materials used in the current study are available at the Open Science Framework (OSF) at https://osf.io/qsa3g/?view_only=fd718de513d744c28a40bec9dda7d073 (accessed on 1 May 2025).

## Notes

1 Time and money differ in that spending money on oneself results in having less money for oneself, whereas spending time on oneself makes one feel that one has more time for the self.

2 Target sample size was determined based on the needs of the larger study.

3 Data were collected in 2019 around the time of the warning that automated bots were found to harm data quality on MTurk (Stokel-Walker, 2018). We therefore tried to ensure data quality by including three attention check items asking participants to select a specific value on Likert scales (e.g., “If you are reading this statement, please select ‘Slightly’ as your answer”) and three items asking participants to select the correct information provided in the vignettes (e.g., “Based on the vignette, what was the task assigned by your supervisor?”). The number of participants who failed our attention check questions was higher than usual because participants needed to correctly answer all six attention check items to be included in the analyses.

4 We do not believe that the period of COVID-19 moderated our main findings, as we conducted our studies before (Study 1), during (Study 2), and at the end of the pandemic (Study 3) and replicated consistent patterns of results. However, the risks of infection during the pandemic may have diminished participants’ willingness to interact with others in Study 2. Indeed, for the same vignette, the average helping time in Study 2 was shorter (*M* = 94.23, *SD* = 52.74) than in Study 3, which was conducted toward the end of the pandemic (*M* = 111.46, *SD* = 67.99), and Study 1, which was conducted before the pandemic (*M* = 119.83, *SD* = 65.99).

5 The Time Perception Scale also included two items assessing the perception of taking time from others (e.g., “I feel that I am taking away other people’s time;” *r* = 0.87) but these items were excluded from analyses because they were irrelevant in the context of helping others.

6 Separate analyses for each item produced the same results as using the combined index for willingness to help.

7 Four participants failed to complete the Time Perception Scale, so analyses were based on *n* = 530. However, their data were included in the main analyses since they completed the manipulation, main dependent variables, and mediators.

## Supplementary Materials

The following supporting information can be downloaded at https://www.mdpi.com/article/10.3390/ejihpe15050090/s1, Table S1: Descriptive statistics of variables in Study 1; Table S2: Descriptive statistics of variables in Study 2; Table S3: Contrast tests of the manipulation checks in Study 3; Table S4: Contrast tests for the main variables in Study 3; Table S5: Correlations of variables in Study 3; Figure S1: Means of time perception by conditions (Study 2); Figure S2: Means of time perception by conditions (Study 3); Figure S3: Prosocial motives as mediators of the association between offering time manipulation and willingness to help; Figure S4: Prosocial motives as mediators of the association between offering time manipulation and helping time.

## Appendix A

Vignette 1 (Study 1)

You are a retail employee working at Hipwear. One day at work, your close friend and colleague, Alex, calls you and tells you that she will be very late to her shift. Alex is the person who takes over your place after your shift is over. You were planning to study for an exam that is coming up for you soon. If you leave right when your shift is over, Alex’s pay will be deducted for the hours she missed. You have 2 h of time to cover for Alex and/or study for your upcoming exam. You decide to allocate _______ minutes of your time to cover for your close friend, Alex.

Vignette 2 (Study 1)

Your close friend, who lives in your neighborhood, is sick. Today, on your day off from work, your friend calls you. Knowing that you have the day off, your friend asks you if you could help him/her pick up some groceries at the grocery store and pick up his/her clothes from the cleaner. You have 2 h of free time today that you can use to clean your house and/or to help your friend out. You decide to allocate _________ minutes of your free time to help your friend with his/her errands.

Vignette 3 (Studies 1, 2, and 3)

You are an office employee in Fundsworth. Earlier last week, you and your colleagues have been tasked by your supervisor to summarize the data from the company’s sales in the previous year. Today, you find yourself completing the given task—one day before the actual deadline. All your colleagues have also completed the task, except for your close friend, Jeff. You can see from your desk that Jeff is struggling with the supervisor’s task. There are 4 h left until the end of the workday. You have 4 h to get ahead of your work so you can go home early on Friday and/or to help Jeff with his task. You decide to allocate ______ minutes of your time to help your close friend, Jeff with his work.
