# Supplementary material for: Nonzero-Sum Time Perception Is Associated with Greater Willingness to Help
Source: Eur J Investig Health Psychol Educ. 2025 May 21;15(5):90. doi: 10.3390/ejihpe15050090 (PMC12110441; doi:10.3390/ejihpe15050090)
Supplement: Supplementary file 1 [file ejihpe-15-00090-s001.zip › ejihpe-3545382-supplementary.pdf]

Nonzero-sum Time Perception is Associated with Greater Willingness to Help

Yu Niiya, Syamil Yakin, Lora E. Park and Ya-Hui Chang

Supplemental Materials

Table S1

Descriptive statistics of variables in study 1 (n = 189)

	M	*SD*	Nonzero-sum time	Offering time	Time-taken-away
Nonzero-sum time	3.19	1.09	--		
Offering time	3.53	1.03	.60**	--	
Time-taken-away	2.62	1.34	.29**	.49**	--
Helping time (in %)	56.19	23.49	.21**	.08	-.02

Note: For helping time, given that the three vignettes had a different amount of available free time (i.e., 2 to 4 hours), we converted the helping time measure of each vignette to percentages and then computed their means. ** $p < .01$

Table S2

Descriptive statistics of variables by conditions in study 2 (n = 471)

	Condition						Total (n = 471)	
	Nonzero-sum Time (n = 153)		Offer Time (n = 166)		Time-taken-away (n = 152)			
	M	*SD*	*M*	*SD*	*M*	*SD*	*M*	*SD*
Nonzero-sum time	3.30	0.72	3.05	0.65	3.07	0.74	3.14	0.71
Offering time	3.52	0.78	3.46	0.74	3.46	0.80	3.48	0.77
Time-taken-away	2.40	0.89	2.68	0.81	2.88	0.86	2.65	0.87
Willingness to help	5.07	1.12	4.78	1.11	4.83	1.06	4.89	1.10
Helping time (in min)	98.37	56.38	94.78	53.06	89.45	48.39	94.23	52.74

Table S3

Contrast tests for the manipulation checks in study 3 (*n* = 530)

Variables	Contrast Tests
	<u>Nonzero-sum time condition as reference group</u>
Nonzero-sum time	Offering time condition: $F(1, 526) = 136.05$, $p < .001$, $\eta_p^2 = .21$
	Time-taken-away condition: $F(1, 526) = 140.53$, $p < .001$, $\eta_p^2 = .21$
	Control group: $F(1, 526) = 218.77$, $p < .001$, $\eta_p^2 = .29$
	<u>Offering time condition as reference group</u>
Offering time	Non-zero sum time condition: $F(1, 526) = 29.99$, $p < .001$, $\eta_p^2 = .05$
	Time-taken-away condition: $F(1, 526) = 3.33$, $p = .07$, $\eta_p^2 = .21$
	Control group: $F(1, 526) = 243.21$, $p < .001$, $\eta_p^2 = .32$
	<u>Time-taken-away condition as reference group</u>
Time-taken-away	Non-zero sum time condition: $F(1, 526) = 215.02$, $p < .001$, $\eta_p^2 = .29$
	Offering time condition: $F(1, 526) = 7.58$, $p = .006$, $\eta_p^2 = .01$
	Control group: $F(1, 526) = 205.14$, $p < .001$, $\eta_p^2 = .28$

Table S4

Contrast tests for the main variables in study 3 (*n* = 534)

Variables	Contrast Tests
	Nonzero-sum time condition as reference group
	Offering time condition: $F(1, 530) = 12.91$, $p < .001$, $\eta_p^2 = .02$
	Time-taken-away condition: $F(1, 530) = 6.01$, $p = .015$, $\eta_p^2 = .01$
Willingness to help	Control group: $F(1, 530) = 0.73$, $p = .39$, $\eta_p^2 = .001$
	Control group as reference group
	Offering time condition: $F(530) = 9.93$, $p = .002$, $\eta_p^2 = .02$
	Time-taken-away condition: $F(1, 530) = 3.51$, $p = .062$, $\eta_p^2 = .01$
	Nonzero-sum time condition as reference group
	Offering time condition: $F(1, 530) = 9.52$, $p = .002$, $\eta_p^2 = .02$
	Time-taken-away condition: $F(1, 530) = 8.13$, $p = .005$, $\eta_p^2 = .02$
Time allocated to help	Control group: $F(1, 530) = 6.10$, $p = .014$, $\eta_p^2 = .01$
	Control group as reference group
	Offering time condition: $F(530) = 0.83$, $p = .36$, $\eta_p^2 = .002$
	Time-taken-away condition: $F(1, 530) = 0.44$, $p = .51$, $\eta_p^2 = .001$

	Nonzero-sum time condition as reference group
	Offering time condition: $F(1, 530) = 5.47$, $p = .020$, $\eta_p^2 = .01$
	Time-taken-away condition: $F(1, 530) = 2.91$, $p = .089$, $\eta_p^2 = .01$
Recipient-enhancement motives	Control group: $F(1, 530) = 6.21$, $p = .013$, , $\eta_p^2 = .01$
	Control group as reference group
	Offering time condition: $F(530) = .002$, $p = .96$, $\eta_p^2 = .000$
	Time-taken-away condition: $F(1, 530) = .04$, $p = .52$, $\eta_p^2 = .001$
	Nonzero-sum time condition as reference group
	Offering time condition: $F(1, 530) = 7.06$, $p = .008$, $\eta_p^2 = .01$
	Time-taken-away condition: $F(1, 530) = 5.25$, $p = .022$, $\eta_p^2 = .01$
Recipient-support motives	Control group: $F(1, 530) = 9.05$, $p = .003$, , $\eta_p^2 = .02$
	Control group as reference group
	Offering time condition: $F(530) = 0.02$, $p = .90$, $\eta_p^2 = .000$
	Time-taken-away condition: $F(1, 530) = 0.27$, $p = .60$, $\eta_p^2 = .001$
	Nonzero-sum time condition as reference group
	Offering time condition: $F(1, 530) = 5.62$, $p = .02$, $\eta_p^2 = .01$
	Time-taken-away condition: $F(1, 530) = 1.54$, $p = .22$, $\eta_p^2 = .003$
Relationship closeness	Control group: $F(1, 530) = 6.22$, $p = .013$, , $\eta_p^2 = .01$
	Control group as reference group
	Offering time condition: $F(530) = 0.01$, $p = .94$, $\eta_p^2 = .000$
	Time-taken-away condition: $F(1, 530) = 1.38$, $p = .24$, $\eta_p^2 = .003$

Table S5

Correlations of variables in study 3 (n = 534)

	1	2	3	4	5	6	7
1. Nonzero-sum time<br>(1 = Nonzero-sum time; 0 = Offering time; 0 = Time-taken-away; 0 = Control)	--						
2. Offering time<br>(1 = Offering time; 0 = Nonzero-sum time; 0 = Time-taken-away; 0 = Control)	-.28**	--					
3. Time taken away<br>(1 = Time-taken-away; 0 = Nonzero-sum time; 0 = Offering time; 0 = Control)	-.37**	-.39**	--				
4. Recipient-enhancement motives	.11**	-.04	-.06	--			
5. Recipient-support motives	.14**	-.04	-.07	.79**	--		
6. Inclusion of others in self	.10*	-.05	-.06	.46**	.41**	--	
7. Willingness to help	.11*	-.14**	.08	.40**	.45**	.19**	--
8. Helping time	.14**	-.07	-.01	.35**	.42**	.25**	.50**

Note. ***p* < .01, **p* < .05

Figure S1

Means of time perception by conditions (study 2)

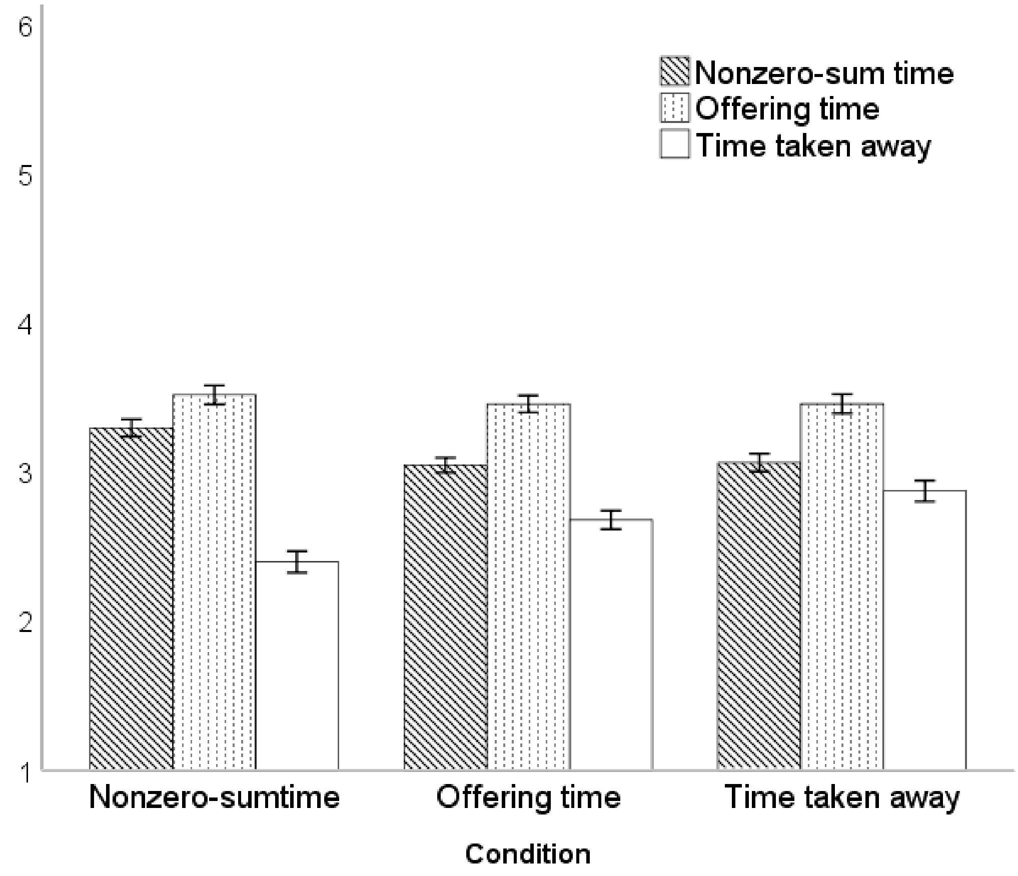

Note: Error bars are +1 and -1 standard errors.

Figure S2

Means of time perception by conditions (study 3)

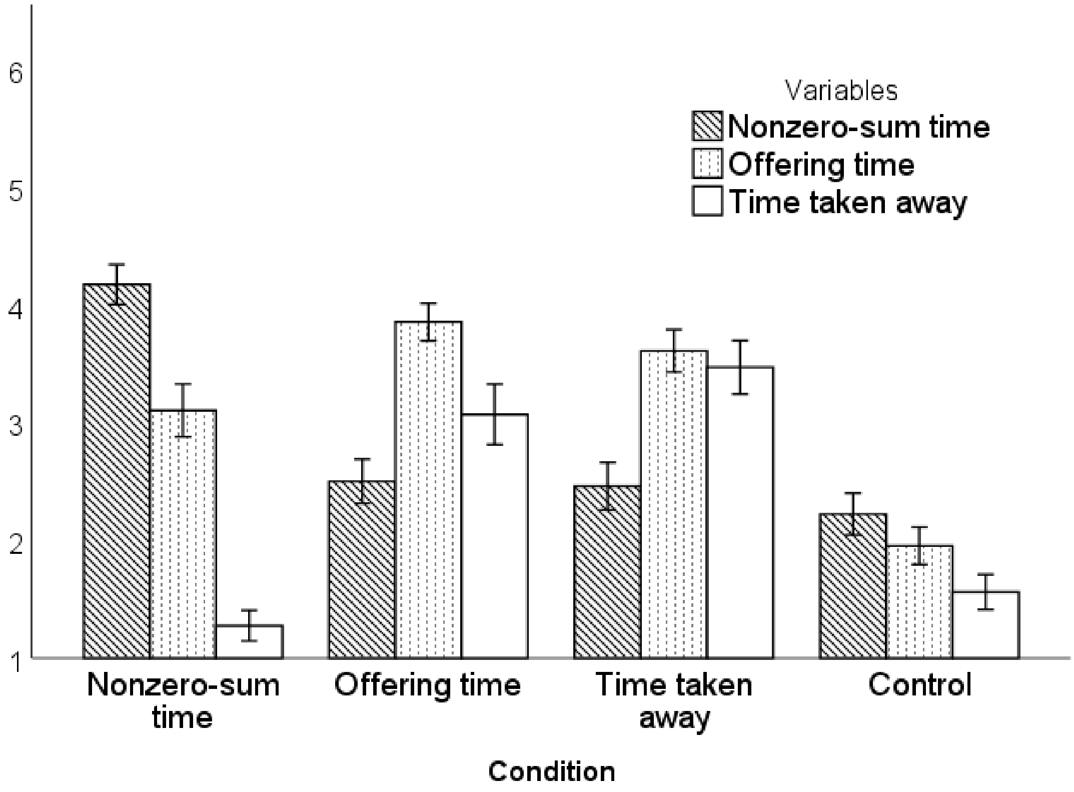

Note: Error bars are +1 and -1 standard errors.

Figure S3

Prosocial motives as mediators of the association between offering time manipulation and willingness to help (study 3)

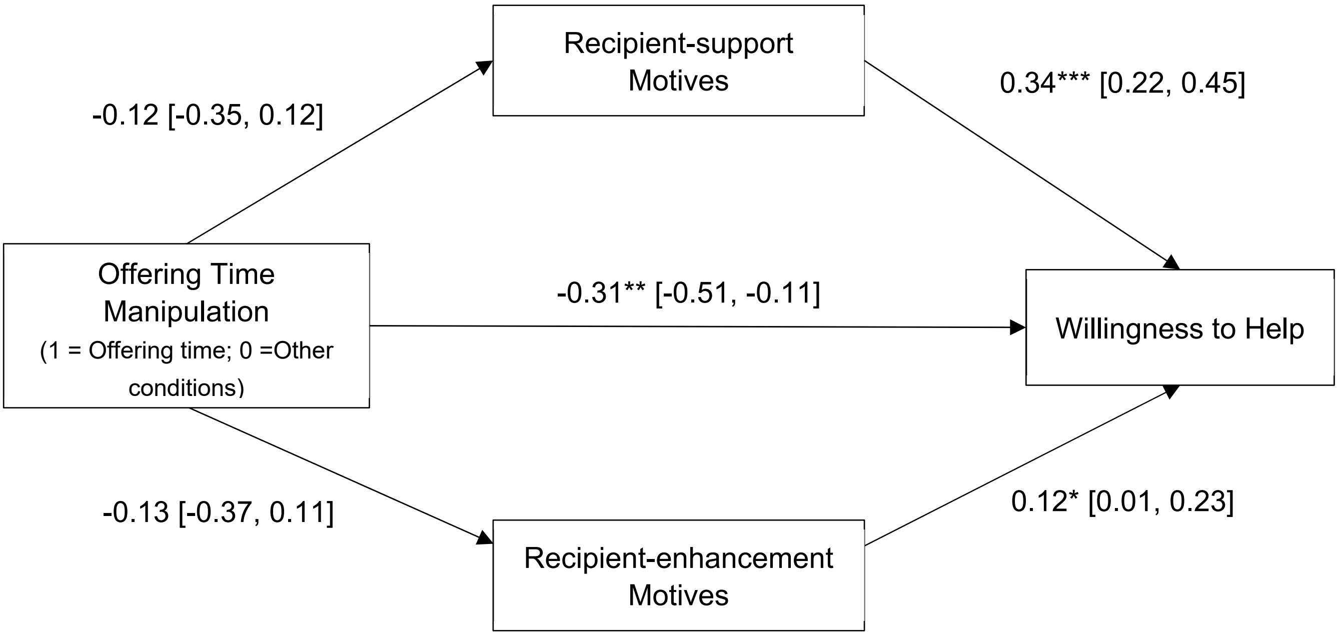

Indirect effect via recipient-support motives: -0.04, SE = 0.04, 95% CI = [-0.13, 0.04]

Indirect effect via recipient-enhancement motives: b = -0.02, SE = 0.02, 95% CI = [-0.06, 0.01]

Note: Offering time manipulation was coded as 1 = Offering time condition, 0 = Nonzero-sum time condition, 0 = Time-taken-away condition, 0 = Control. Values are unstandardized coefficients. * $p < .05$, ** $p < .01$, *** $p < .001$.

Figure S4

Prosocial motives as mediators of the association between offering time manipulation and helping time (study 3)

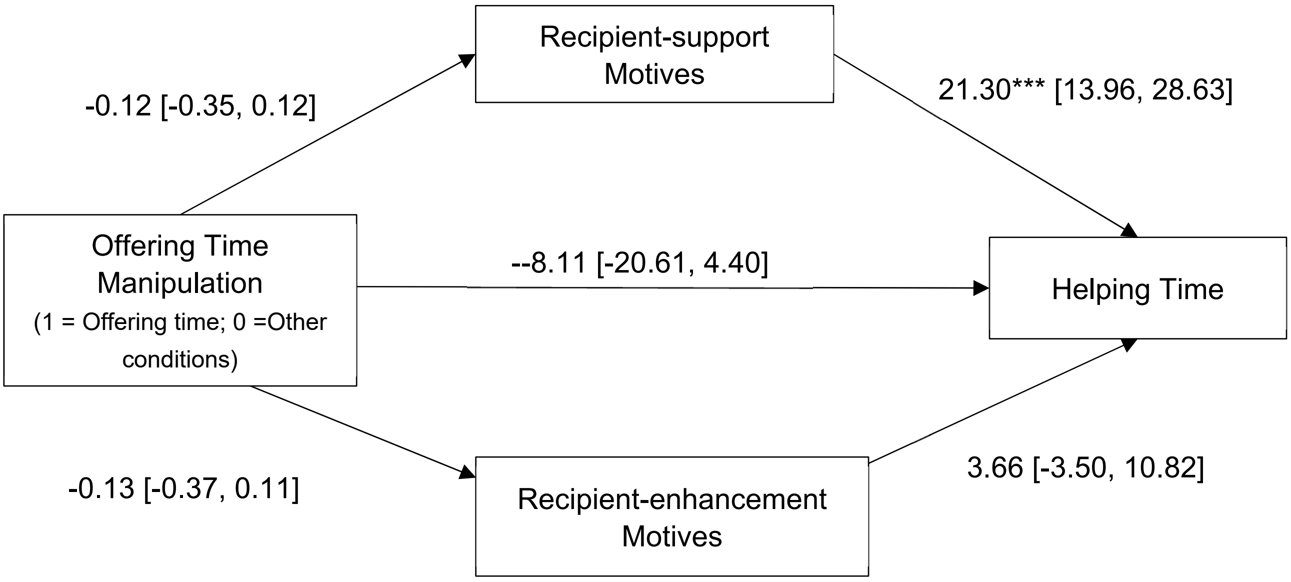

Indirect effect via recipient-support motives: -2.45, SE = 2.48, 95% CI = [-7.60, 2.13]

Indirect effect via recipient-enhancement motives: b = -0.47, SE = 0.79, 95% CI = [-2.36, 0.88]

Note: Nonzero-sum manipulation was coded as 1 = Nonzero-sum time condition, 0 = Offering time condition, 0 = Time-taken-away condition, 0 = Control. Values are unstandardized coefficients. ***p < .001.
